# The chromosome 6q22.33 region is associated with age at diagnosis of type 1 diabetes and disease risk in those diagnosed under 5 years of age

**DOI:** 10.1007/s00125-017-4440-y

**Published:** 2017-10-05

**Authors:** Jamie R. J. Inshaw, Neil M. Walker, Chris Wallace, Leonardo Bottolo, John A. Todd

**Affiliations:** 1 JDRF/Wellcome Trust Diabetes and Inflammation Laboratory, Wellcome Trust Centre for Human Genetics, University of Oxford, NIHR Oxford Biomedical Research Centre, Nuffield Department of Medicine, Roosevelt Drive, Oxford, OX3 7BN UK; 2 JDRF/Wellcome Trust Diabetes and Inflammation Laboratory, Cambridge Institute for Medical Research, University of Cambridge, Cambridge, UK; 3 Clinical Informatics, Cambridge University Hospitals NHS Foundation Trust, Cambridge, UK; 4 Department of Medicine, University of Cambridge, Cambridge, UK; 5 MRC Biostatistics Unit, Cambridge Institute of Public Health, Cambridge, UK; 6 Department of Medical Genetics, University of Cambridge, Cambridge, UK; 7 The Alan Turing Institute, London, UK

**Keywords:** Age at diagnosis, Early diagnosis, Genetic risk, Type 1 diabetes

## Abstract

**Aims/hypothesis:**

The genetic risk of type 1 diabetes has been extensively studied. However, the genetic determinants of age at diagnosis (AAD) of type 1 diabetes remain relatively unexplained. Identification of AAD genes and pathways could provide insight into the earliest events in the disease process.

**Methods:**

Using ImmunoChip data from 15,696 cases, we aimed to identify regions in the genome associated with AAD.

**Results:**

Two regions were convincingly associated with AAD (*p* < 5 × 10^−8^): the MHC on 6p21, and 6q22.33. Fine-mapping of 6q22.33 identified two AAD-associated haplotypes in the region nearest to the genes encoding protein tyrosine phosphatase receptor kappa (*PTPRK*) and thymocyte-expressed molecule involved in selection (*THEMIS*). We examined the susceptibility to type 1 diabetes at these SNPs by performing a meta-analysis including 19,510 control participants. Although these SNPs were not associated with type 1 diabetes overall (*p* > 0.001), the SNP most associated with AAD, rs72975913, was associated with susceptibility to type 1 diabetes in those individuals diagnosed at less than 5 years old (*p* = 2.3 × 10^−9^).

**Conclusion/interpretation:**

*PTPRK* and its neighbour *THEMIS* are required for early development of the thymus, which we can assume influences the initiation of autoimmunity. Non-HLA genes may only be detectable as risk factors for the disease in individuals diagnosed under the age 5 years because, after that period of immune development, their role in disease susceptibility has become redundant.

**Electronic supplementary material:**

The online version of this article (10.1007/s00125-017-4440-y) contains peer-reviewed but unedited supplementary material, which is available to authorised users.

## Introduction

Since the introduction of genome-wide association studies (GWAS), over 50 regions in the genome have been associated with susceptibility to type 1 diabetes [1–6], but less research has examined the genetic determinants of age at diagnosis (AAD) of type 1 diabetes. One study, limited to specific SNPs in regions associated with type 1 diabetes, identified that the MHC, IL-2 (*IL2*) and renalase (*RNLS*) gene regions showed evidence of association with AAD [7, 8]. However, the question has never been examined in a genome-wide fashion. Identification of genes associated with the initiation of the anti-islet autoimmunity, which is in most cases established by the age of 3 years [9], could help to establish the earliest events in the disease process.

Here we aimed to identify genetic determinants of AAD in a more powerful approach using data from an extensive SNP panel, the custom array ImmunoChip [4], by combining data from independent cases and affected sib-pairs (ASPs) to increase sample size and improve the genetic map through imputation.

## Methods

We analysed data from six cohorts, independent cases from the UK Genetic Resource Investigating Diabetes (GRID) cohort [2], the Northern Irish GRID (NI) cohort (used here for the first time), and the Finnish IDDMGEN (Tyypin 1 Diabetekseen Sairastuneita Perheenjäsenineen) and T1DGEN (Tyypin 1 Diabeteksen Genetiikka) cohorts [10], in addition to ASPs from the Type 1 Diabetes Genetics Consortium (T1DGC) cohort [11] (from north America, Europe, Asia and the UK) and the UK Warren cohort [12]. The majority of individuals were diagnosed in childhood, with 92% diagnosed at less than 20 years of age. Genotyping (see electronic supplementary material [ESM] Genotyping) was performed on 16,015 affected individuals, 8683 (54%) independent participants and 7332 (46%) ASPs (Table 1). Quality control was performed prior to analysis to minimise the risk of reporting false-positive results (ESM Quality control, ESM Figs 1–3).

**Table 1:** Baseline characteristics and inclusion in the primary AAD analysis after quality control

Cohort	Country	Type	Genotyped	Included	AAD: median (IQR)	SNPs after QC
GRID	UK	Independent	6799	6736	8 (4, 11)	164,953
IDDMGEN	Finland	Independent	1111	1073	9 (5, 12)	156,343
NI	NI	Independent	524	509	7 (4, 10)	156,343
T1DGEN	Finland	Independent	249	249	16 (10, 24)	156,343
Warren	UK	ASP	907	839	10 (5, 15)	156,343
T1DGC	Asia	ASP	960	919	10 (5, 14)	167,537
T1DGC	Europe	ASP	2521	2485	11 (6, 17)	167,537
T1DGC	USA	ASP	2593	2544	8 (4, 13)	167,537
T1DGC	UK	ASP	351	342	8 (4, 11)	167,537
Total	All	All	16,015	15,696	9 (5, 12)	150,381

The intersect of the SNPs that passed QC across genotype batch were included in the analysis. Total SNPs after QC refers to the common set of SNPs across all collections

QC, quality control

### SNP imputation

Where mentioned, we used IMPUTE2 software (http://mathgen.stats.ox.ac.uk/impute/impute_v2.html) [13, 14], using 1000 Genomes Project data [15] (version III) as the reference dataset (https://mathgen.stats.ox.ac.uk/impute/1000GP_Phase3.html) to perform SNP imputation. We excluded SNPs with a minor allele frequency of less than 0.01 or an imputation information score less than 0.8 as a quality control measure (an imputation score of 0 indicates no certainty in the imputed genotype, whereas a score of 1 indicates no uncertainty in the imputed genotype).

### Association discovery using ImmunoChip data

As we had a population that comprised related and independent cases, from a variety of cohorts containing individuals from multiple countries between and within cohorts, it was crucial to account for population structure, to avoid reporting spurious associations. We did this by performing an inverse-variance weighted meta-analysis [16], in which we stratified the samples by cohort and examined the effect of each SNP on log_e_ AAD (assuming an additive mode of inheritance), adjusting for sex and the top five principal components within the cohort to account for population structure.

However, in cohorts with related individuals, standard principal components analysis may not correctly identify the population structure, as the population-level clusters are confounded by the relatedness between individuals. Therefore, in these cohorts, we used principal components analysis in related-samples, PC-AiR [17], which estimates principal components by identifying a subset of genetically dissimilar individuals and performs principal components analysis on this subset, before using these principal components to estimate the ancestry of the remaining individuals in the cohort. This was performed using the GENESIS R package (https://www.bioconductor.org/packages/devel/bioc/html/GENESIS.html, version 2.2.7) [18]. We applied a variance-components model using the GenABEL R package (http://www.genabel.org/packages/GenABEL, version 1.8-0, Grammar-Gamma method) [19] to analyse the effect of each SNP in cohorts of related individuals, which takes into account relatedness between individuals. ESM Fig. 4 provides a schematic overview of the association discovery meta-analysis procedure.

We also performed the association discovery analysis using an alternative approach. First, we fitted a linear mixed model [20] with the log_e_ AAD as the outcome, adjusting for sex as a fixed effect and including random effects for cohort, country and family identifier. We then used the residuals from the linear mixed model as the outcome variable and tested the association of each SNP using a linear regression model. We called this approach the ‘residual-based model’, and it has been proposed by Aulchenko et al [21] to make genome-wide analysis for related individuals possible. Since our aim was eventually to fine-map statistically significant regions, the advantage of the residual-based model is that the residuals can also be used as the outcome variable in a fine-mapping analysis for continuous traits. ESM Fig. 5 illustrates the steps of this second approach used for variants discovery.

SNPs were declared to be associated with AAD if the *p* value was less than a genome-wide significance threshold of 5 × 10^−8^. We also highlight regions associated with a false discovery rate (FDR) [22] of less than 0.05, to identify the regions next most likely to influence AAD. In this analysis, we used a stringent definition, removing the MHC region before calculating the FDR, as including all the highly associated SNPs from the MHC can inflate the threshold below which SNPs are declared to be associated, increasing the probability of reporting false-positive results.

To add further evidence to the detected associated SNPs, we combined cases with 19,510 control individuals (ESM Table 1) and performed an inverse-variance weighted meta-analysis across cohorts, examining the effect of the SNPs on risk of type 1 diabetes overall and in those who were diagnosed at less than 5 years of age. Cohorts of independent individuals were analysed by fitting a logistic regression model adjusting for the top five principal components and examining the effect of the SNP of interest on risk of type 1 diabetes. Cohorts of related individuals were analysed using a generalised linear mixed model association test [23], using the GMMAT R package (https://www.hsph.harvard.edu/han-chen/software/, version 0.7-1), adjusting for the top five principal components as fixed effects, and using a kinship matrix to define the covariance structure of the random effect included in the model. We present ORs for the SNPs associated with AAD for their association with type 1 diabetes overall and for those diagnosed at less than 5 years of age, to compare the direction of effect between analyses, with a consistent direction of effect adding further evidence that the association was genuine.

### Association discovery: imputed data

A subset of 1768 cases from the GRID cohort had data that had been genotyped using the Affymetrix GeneChip Mapping 500K, and 3833 had been genotyped using the Illumina 550K Infinium microarray platform [3], both of which are GWAS chips and cover a broader spectrum across the genome than the ImmunoChip. We stratified the GRID cohort into strata, one containing cases who had been genotyped using Affymetrix technology, and the other containing cases who had been genotyped using Illumina. We imputed in 1 Mb blocks across the entire genome, and then tested the association of each SNP with log_e_ AAD for both strata using SNPTEST software (version II, using the frequentist option, https://mathgen.stats.ox.ac.uk/genetics_software/snptest/snptest.html) [24], before combining results by an inverse-variance weighted, fixed-effects meta-analysis. Owing to an uncertain genotype call, we used the expectation maximisation option to estimate the association of each SNP while performing the imputation of the missing genotypes.

### Fine-mapping

In AAD-associated regions (*p* < 5 × 10^−8^), we imputed SNPs to obtain the densest SNP set possible in that region, and performed fine-mapping to identify groups of candidate causal SNPs. We used GUESSFM (https://github.com/chr1swallace/GUESSFM version 1.0.1), [25] a fine-mapping algorithm [26, 27] that allows more than one causal SNP in the region to be identified. Briefly, GUESSFM identifies models, that is, combinations of SNPs, with high posterior support given the likelihood and the priors, by carrying out a stochastic search. Models are ranked by the frequency with which they appear in the search. The SNPs included in the most visited models are most likely to be causally associated with AAD. In contrast to other fine-mapping methods that use summary statistics, GUESSFM makes use of raw genotype data. One output from GUESSFM is the group marginal posterior probability of inclusion (gMPPI), which groups SNPs in tight linkage disequilibrium (LD) and can be thought of as the posterior support that exactly one of the SNPs in the group is causal.

The outcome variable was the same set of residuals used in the association discovery analysis residual-based model, so population and family structure had been accounted for. Our primary analyses used the default prior for the expected number of causal variants in the region to be three. For reproducibility of the results, we examined also the effect of changing the expected number of causal SNPs to two or six. For comparison, we fitted stepwise linear regression models in each region of interest (with the same set of residuals as the outcome), initially including the most significant SNP in the region, followed by the most associated SNP conditional on the initial SNP. We repeated this process until one of the SNPs in the model fell below a commonly used [25, 28] significance threshold of *p* = 5 × 10^−6^.

### Haplotype and diplotype analyses

The output from GUESSFM identifies groups of candidate causal SNPs in LD that are most likely to be associated with the phenotype. We examined these groups detected by GUESSFM in a haplotype analysis to highlight transmission patterns and to visualise haplotype membership of the selected SNPs. We phased SNPs using SNPHAP software (https://github.com/chr1swallace/snphap, version 1.3), which generates the posterior probability of each haplotype for each individual. To capture the uncertainty of the haplotype phase, we simulated ten haplotype datasets, where, in each dataset, the haplotype for every individual was sampled from their haplotype posterior distribution. The effect size of each haplotype relative to the most common was calculated in each dataset using a linear mixed model, with log_e_ AAD as the outcome, adjusting for haplotype and sex as fixed effects, and family identifier, cohort and country as random effects. Results were pooled across the ten datasets using the mice R package (http://www.jstatsoft.org/v45/i03/, version 2.9) for combining results from multiply imputed datasets [29]. Once haplotypes had been estimated, we extended the analysis to examine diplotypes by combining the haplotypes from the two chromosomes and using the methods described above to estimate the effect of each diplotype.

All analyses were carried out using R version 3.3.2.

All samples were collected after approval from the relevant research ethics committees, and written informed consent was obtained from the participants.

## Results

### Association discovery ImmunoChip analysis

**Fig. 1:**
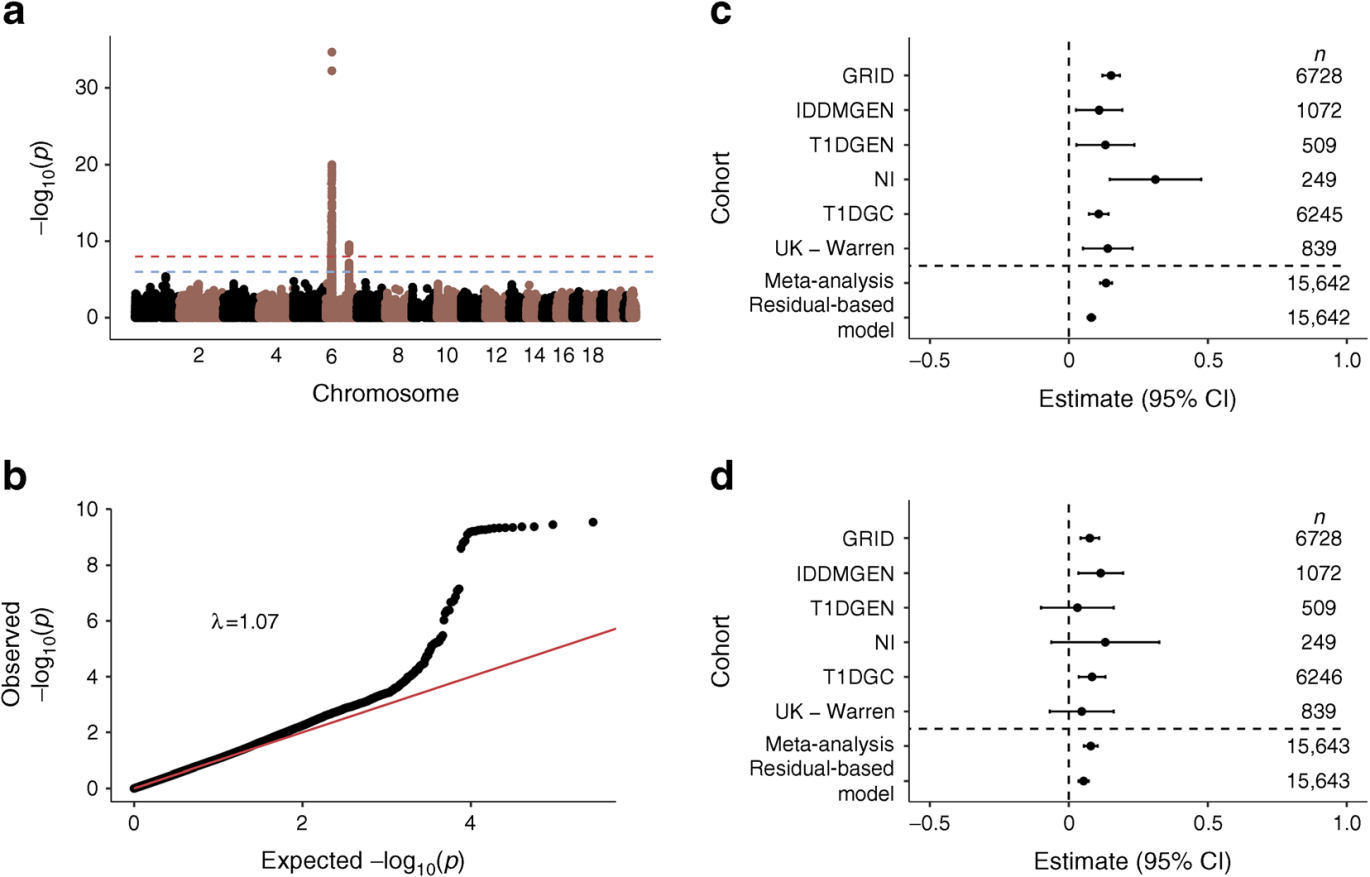
(**a**) Manhattan plot from the association discovery meta-analysis for AAD of type 1 diabetes. (**b**) Quantile–quantile plot (excluding the MHC region). (**c**) Forest plot for the lead SNP in the MHC region, rs9273363. (**d**) Forest plot for the lead SNP in the 6q22.33 region, rs72975913

Results from the meta-analysis identified two regions that were associated at *p* < 5 × 10^−8^, the MHC region and the 6q22.33 region, the latter of which contains the genes encoding protein tyrosine phosphatase receptor kappa (*PTPRK*) and thymocyte-expressed molecule involved in selection (*THEMIS*) (Fig. 1). The index SNP in the MHC was rs9273363 (*p* = 2.16 × 10^−35^), which has previously been shown to be associated with type 1 diabetes [30], to tag the HLA DQB1*03:02 genotype [31] and to be located in a potential enhancer region of the major type 1 diabetes gene, *HLA-DQB1* [32].

In the chromosome 6q22.33 region, the lead SNP was rs72975913 (*p* = 2.94 × 10^−10^), which is in LD (*r* ^2^ = 0.99) with a key SNP associated with coeliac disease (rs72975916) [33]. Results from the residual-based model were similar, with the same two regions, and no others, reaching genome-wide significance. Manhattan and quantile–quantile plots for the residual-based model can be found in ESM Fig. 6, and residual plots for this analysis in ESM Figs 7 and 8.

The results from the residual-based model estimate that the addition of an A allele (the minor allele in controls) at the lead MHC SNP, rs9273363, is associated with an 11.8% decrease in AAD, which translates to a decrease of 1.11 years (13 months). The addition of the major C allele at the lead 6q22.33 SNP, rs72975913, is associated with a younger AAD by 0.7 years (8 months). If an individual is homozygous for the AAD risk allele at the lead SNP in both regions, they are estimated to be diagnosed 4.12 years younger than those who are homozygous for the non-risk allele at both loci.

Although there were just two regions that reached genome-wide significance, Table 2 shows that region 1q24.3, which contains the Fas ligand (*FASLG*) gene, contains at least one SNP with some evidence (FDR <0.05) of association with AAD. It adds weight to the evidence that this SNP might be truly related to AAD since the type 1 diabetes risk effect direction in those diagnosed at less than 5 years of age is the same as the AAD effect direction for each SNP, that is, the risk allele for type 1 diabetes is associated with younger AAD.

**Table 2:** Regions with evidence (FDR <0.05) of association with AAD of type 1 diabetes

Region	Lead SNP	Risk < major allele	Nearest gene(s)	Estimated % change in AAD (95% CI) from linear mixed model	Meta-analysis, *p* value	Residual-based, *p* value	Region associated with autoimmune diseases?	Overall type 1 diabetes OR (95% CI)	Type 1 diabetes under age 5 years, OR (95% CI)
1q24.3	rs10912265	T < C	*FASLG*	0.06 (0.04, 0.08)	4.0 × 10^−6^	1.2 × 10^−6^	CEL, CRO, T1D	0.95 (0.90, 0.99)	0.82 (0.76, 0.88)
6q22.33	rs802719	C < T	*PTPRK*, *THEMIS*	−0.06 (−0.08, −0.04)	7.0 × 10^−8^	5.6 × 10^−9^	CEL, CRO, MS	1.01 (0.98, 1.05)	1.14 (1.07, 1.20)
6q22.33	rs72975913	A < C	*PTPRK*, *THEMIS*	0.08 (0.05, 0.10)	2.9 × 10^−10^	7.4 × 10^−9^	CEL, CRO, MS	0.92 (0.88, 0.97)	0.78 (0.72, 0.85)
6p21.32	rs9273363	A < C	*HLA-DRB1*, *HLA-DQB1*	−0.12 (−0.10, −0.14)	2.2 × 10^−35^	2.09 × 10^−32^	Multiple	3.52 (3.37, 3.67)	5.03 (4.67, 5.42)

Estimates are for addition of a minor allele. The 6q22.33 region contains two associations: the lead SNP from the residual-based model and the lead SNP from the meta-analysis

CEL, coeliac disease; CRO, Crohn’s disease; MS, multiple sclerosis; T1D, type 1 diabetes

### Association discovery analysis—imputed data

In total, 7,476,246 SNPs were examined for their association with AAD. Just one region reached genome-wide significance, the MHC region (ESM Fig. 9), which indicates that there was not sufficient power to detect associated regions outside the ImmunoChip due to a smaller sample size in this analysis (5601 vs 15,696 in the ImmunoChip analysis).

### Fine-mapping

It is beyond the scope of the current study to consider fine-mapping of the MHC region, and it is well established that the *HLA-DQB1*, *HLA-DRB1*, *HLA-A* and *HLA-B* genes are the primary determinants of the MHC risk in type 1 diabetes [30].

**Fig. 2:**
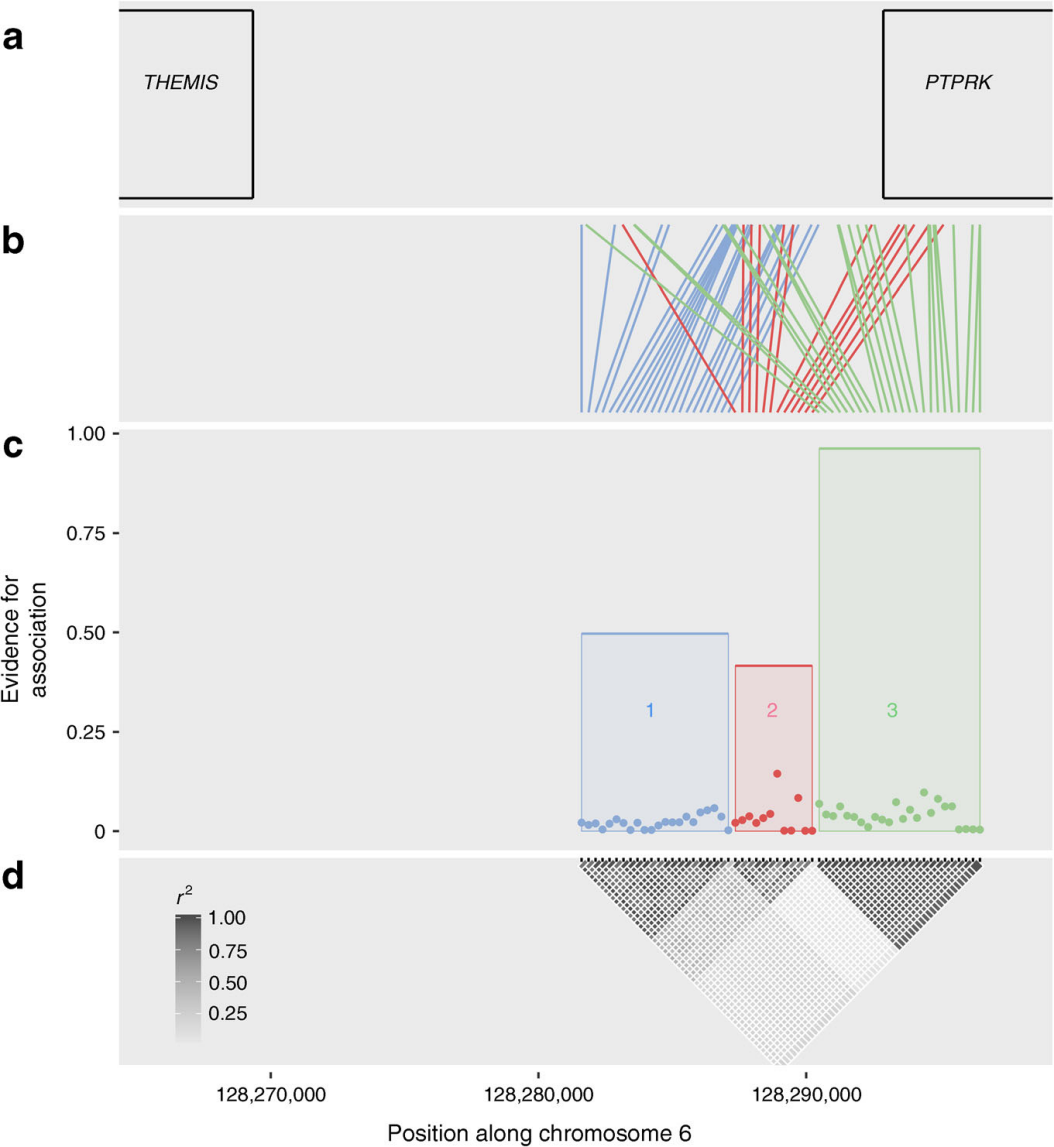
Output from GUESSFM fine-mapping the 6q22.33 region. (**a**) Location of the *PTPRK* and *THEMIS* genes, the closest genes to the candidate causal SNPs. (**b**) Map of the candidate causal SNPs to their physical location along chromosome 6. (**c**) The dots depict the strength of association (marginal posterior probability of inclusion) for each SNP, while the height of the shaded region is the gMPPI, the probability that one of the SNPs in the group is causal for AAD. It shows three signals in the region, termed groups 1 (blue), 2 (red) and 3 (green). (**d**) LD between SNPs

In the 6q22.33 region (positions 127,952,182 to 128,340,790 on chromosome 6, NCBI build 37, https://www.ncbi.nlm.nih.gov), we performed SNP imputation with a concordance of 97.9% (i.e. for each SNP of known genotype, the imputed genotype matched the known genotype at least 97.9% of the time). In total, 786 SNPs were analysed in this region, including 319 imputed SNPs. The GUESSFM analysis highlighted three potential signals in the region (Fig. 2, ESM Table 2). Group 1 comprised 22 SNPs, had a gMPPI of 0.50 and was in LD (*r* ^2^ = 0.49) with group 2, which contained 12 SNPs and had a gMPPI of 0.42; this group contains rs802719, the SNP with the highest posterior support (SNP marginal posterior probability of inclusion = 0.14). Finally, group 3, with the strongest signal, contained 24 SNPs and had a gMPPI of 0.96, including the index SNP from the association discovery analysis, rs72975913. However, the LD between groups 1 and 2 implies that these groups are probably the same signal; in addition, the fact that the SNP with the highest posterior probability is contained in group 2 rather than group 1 implies that the true signal is more likely to be from group 2. The results were similar when changing the prior number of SNPs expected in the model to two or six, both showing three signals in the region. Results from the stepwise linear regression approach indicated that there were two signals: the lead SNP once imputed data was included was SNP rs11753289, which is contained in group 3 from the GUESSFM analysis, with a borderline second signal, from SNP rs802719 (*p* = 7.5 × 10^−6^), which is contained in group 2.

### Haplotype and diplotype analyses

**Fig. 3:**
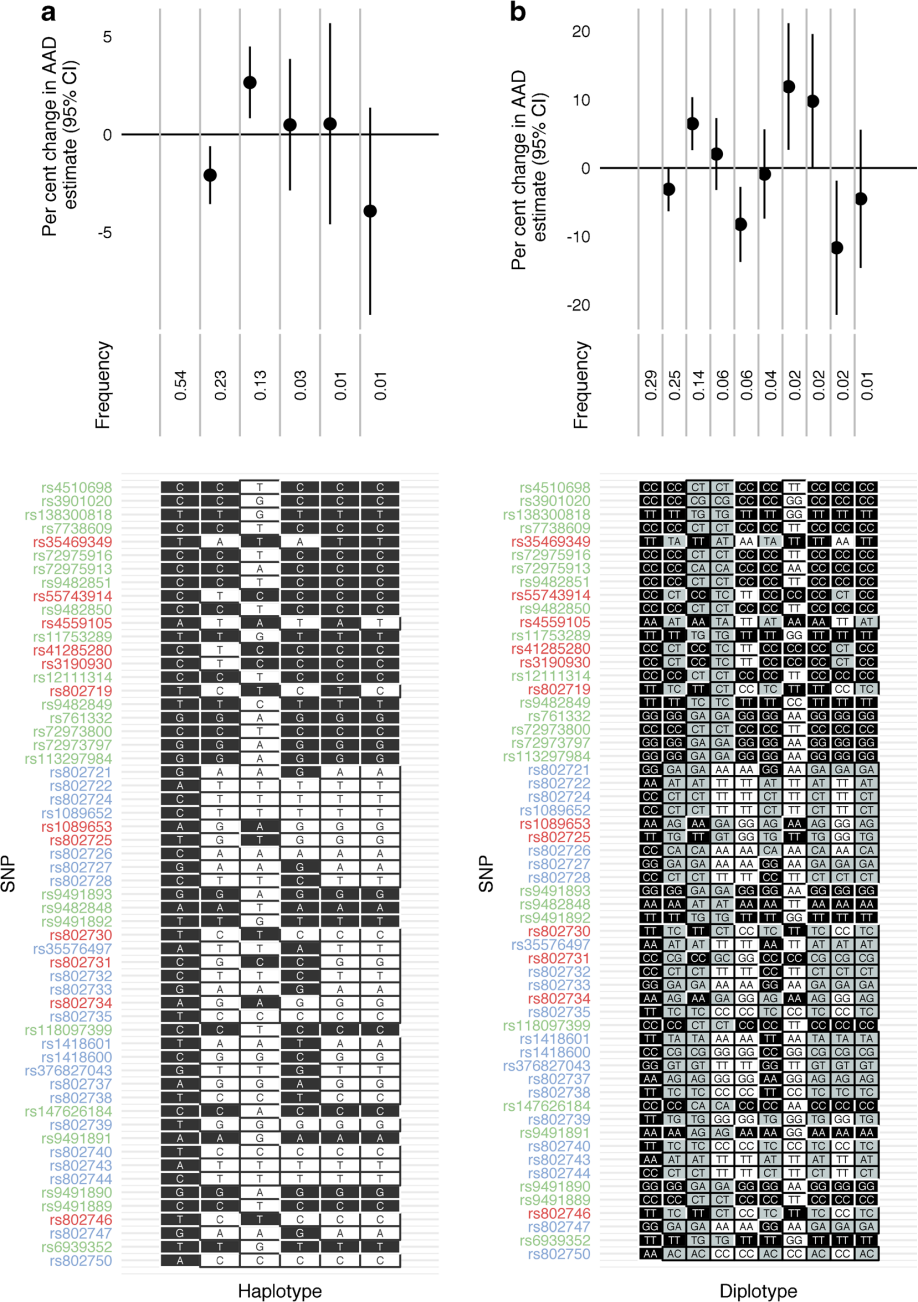
(**a**) Haplotype analysis of the 6q22.33 region with respect to the AAD of type 1 diabetes using SNPs highlighted from the GUESSFM analysis. SNPs are colour-coded according to GUESSFM group 1 (blue), 2 (red) and 3 (green). Black, major alleles; white, minor alleles. (**b**) Diplotype analysis of the same region. Black, major homozygotes; grey, heterozygotes; white, minor homozygotes

In the 6q22.33 region, we examined the 58 SNPs contained in groups 1, 2 and 3 from the GUESSFM analysis and estimated the effect of each haplotype relative to the most common, which was the major allele at each of the 58 SNPs. The minor allele haplotype from the group 2 SNPs is associated with younger AAD, while the minor allele haplotype from the group 3 SNPs is associated with an older AAD (Fig. 3). The LD between groups 1 and 2 can be observed, and the two main signals in the region appear to be from groups 2 and 3. Extending the analysis to examine diplotypes showed that being heterozygous at group 2 SNPs leads to an estimated 3.1% decrease in AAD relative to the most common diplotype, which translates to an estimated younger AAD of 3.1 months. Having two copies of the minor group 2 SNPs leads to an estimated younger AAD of 8.3%, which translates to 8.1 months. In contrast, individuals with one copy of the minor group 3 haplotype have an estimated AAD 6.4% (6.8 months) older than those with the most common diplotype, which increases to 11.9% (12.9 months) if they have two copies of the minor group 3 haplotype (Fig. 3). These results show there are two main risk haplotypes that predispose to younger AAD in the region: the minor allele haplotype for group 2 and the major allele haplotype for group 3.

### Type 1 diabetes risk analysis

We examined one SNP from each of groups 2 and 3 (rs802719 and rs72975913, respectively) from the fine-mapping analysis and examined the effect of these SNPs on type 1 diabetes risk in a meta-analysis including the control participants from ESM Table 1. As expected from the lack of previously reported associations in this region, neither of the SNPs was strongly associated with type 1 diabetes overall (*p* > 0.001) (forest plots are shown in ESM Fig. 10).

**Fig. 4:**
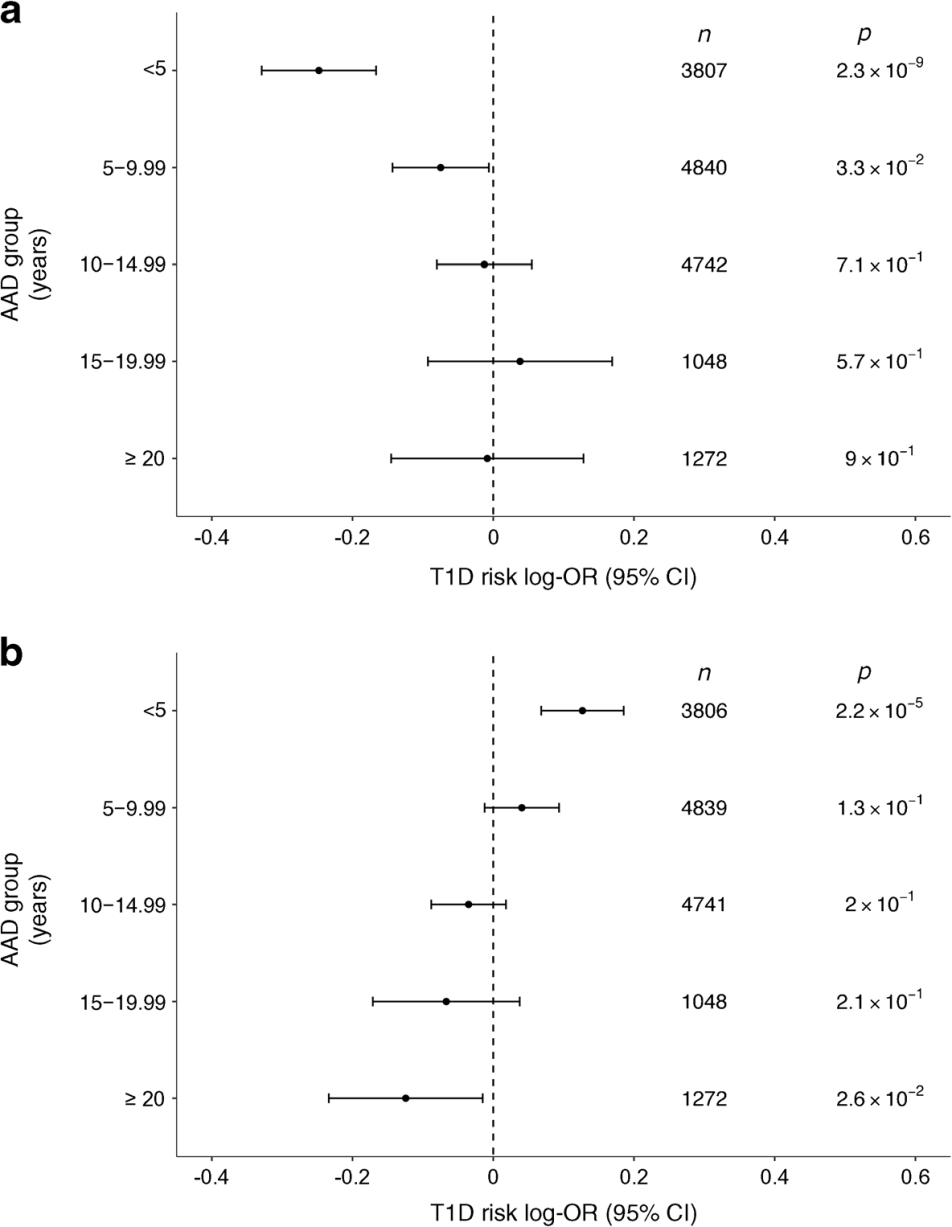
(**a**) Risk of type 1 diabetes (T1D) at SNPs contained in group 3 (GUESSFM analysis) rs72975913, stratified by AAD group. (**b**) Risk of type 1 diabetes at the SNPs contained in group 2, rs802719, stratified by AAD group. The effect size is for addition of a minor allele at the loci, assuming an additive mode of inheritance on the log-odds scale

However, owing to the association with AAD, we stratified each cohort into age group at diagnosis (0–4.99, 5–9.99, 10–14.99, 15–19.99 and ≥20 years) and assessed the association of both SNPs with risk of type 1 diabetes within strata. A strong association was observed in the youngest AAD category at rs72975913 (*p* = 2.3 × 10^−9^, OR 0.78, *n* = 3807) and evidence of an association at rs802719 (*p* = 2.2 × 10^−5^, OR 1.14, *n* = 3806), with the forest plots shown in ESM Fig. 11. The effect estimate was in the same direction as the AAD analysis, with the minor A allele associated with decreased disease susceptibility in those diagnosed at less than 5 years old at rs72975913, which is associated with an older AAD, and for rs802719 the minor C allele increases disease susceptibility and decreases AAD. To examine the sensitivity of our results to the cut-off for which to define the youngest AAD strata, we repeated the analysis, defining the youngest age category first as <4 years and then as <6 years. The results did not change significantly, with rs72975913 remaining associated on a genome-wide basis regardless of cut-off and with a similar effect size observed at rs802719 (ESM Table 3). There is a trend showing how the effect size on disease susceptibility decreases at both SNPs as the age group at diagnosis increases, indicating that the genetic effects in this region act in those diagnosed at a young age (Fig. 4, ESM Figs 12 and 13 for minor allele frequencies). This may explain why this region has not previously been associated with type 1 diabetes as, typically, GWAS datasets include cases who have been diagnosed across a range of ages.

## Discussion

In the first ImmunoChip analysis examining the AAD of type 1 diabetes, we found, as expected, that the MHC was the major genetic influence, while the 6q22.33 region was a second associated region. We performed fine-mapping across the 6q22.33 region, which contains the adjacent *PTPRK* and *THEMIS* genes, and has never previously been associated with AAD [7, 8] or type 1 diabetes, but has been reported to be associated with susceptibility to other autoimmune diseases [33–35].

We identified two haplotypes in the region that were associated with younger AAD. These haplotypes showed evidence of influencing type 1 diabetes susceptibility in those diagnosed at less than 5 years of age but not for other age groups. This implies that the region impacts on risk of type 1 diabetes at a young age but not once the immune system is more fully developed. In an era of increasing sample sizes, this study highlights the benefit of refining a phenotype in order to identify SNPs associated with a subset of individuals who develop a disease. In this case, we highlight a region associated with early-diagnosed type 1 diabetes, but this approach can be applied to heterogeneous diseases to more accurately identify the main genetic determinants in a particular subset. Analyses of the immunology and pancreas histology of type 1 diabetes do reveal distinct autoimmune features in children diagnosed under age 5 years [36]. Genetic findings such as ours will help to identify the key cells and tissues involved, pointing, in this case, to the thymus being particularly important in early, aggressive disease.

*PTPRK* and *THEMIS* are both important for transition of double-positive (CD4^+^, CD8^+^) thymocytes to single-positive thymocytes [37, 38], and there is a reduction in number of mature CD4^+^ T cells in mice that have both genes knocked out, over and above the effect that each gene has independently, indicating that they are both vital to thymopoiesis [39]. Chromosome conformation capture analyses have identified the *PTPRK* promoter as a target of disease-associated sequences [40], supporting its candidacy as the causal gene for AAD.

The 6q22.33 region has been associated with other autoimmune diseases: the index SNP for coeliac disease is rs55743914 [33], which is contained in group 2 in our analysis, and the minor allele is associated with increased risk of coeliac disease and also younger AAD for type 1 diabetes. The secondary signal for coeliac disease (rs72975916) is contained in group 3, and the minor allele is associated with reduced risk of coeliac disease and older AAD for type 1 diabetes, so the directions of effect between AAD of type 1 diabetes and coeliac disease are consistent.

However, the lead SNP for multiple sclerosis, rs802734 [35], which is contained in group 2 in this analysis, has the opposite direction of effect (the minor allele being protective against multiple sclerosis). This signal in multiple sclerosis was not replicated in a larger ImmunoChip analysis [41], and hence the risk of multiple sclerosis at this SNP may also depend on another factor, for example age at onset. Just one signal was detected in the region as associated with Crohn’s disease, rs9491891 [42], which is contained in group 3, with a direction of effect also opposite to that for type 1 diabetes and coeliac disease. The age at onset of multiple sclerosis and Crohn’s disease in the cohorts in these analyses is older than the AAD of the type 1 diabetic individuals in our dataset, while there can be a long delay in coeliac disease between onset and diagnosis [43], so it may be that the difference in effect direction could be to do with a changing immune system with age.

Another possibility is that the SNPs affecting early-diagnosed type 1 diabetes are affecting a different pathway, tissue or cell type from the same SNPs that have the opposite effect in multiple sclerosis and Crohn’s disease; that is, an increased level of a protein in one cell type might increase the risk of type 1 diabetes, while the increase in that same protein in a different cell type might protect against multiple sclerosis or Crohn’s disease. There is no evidence that the 6q22.33 region is associated with age at onset of multiple sclerosis or Crohn’s disease, although there are very few individuals in these analyses who were diagnosed in childhood [35, 42] and it is difficult to assess in coeliac disease given the time between onset and diagnosis [43]. We hypothesise that there is co-localisation in the region between AAD of type 1 diabetes and coeliac disease, given the similar genetic risk variants and also the fact that individuals diagnosed with type 1 diabetes at a young age are more likely to have coeliac disease [44]. Our analysis offers a genetic explanation for this phenomenon.

There was some evidence (FDR <0.05) of an association with AAD at one other region, 1q24.3. This region contains the *FASLG* gene and has been shown to be associated with type 1 diabetes itself [5]; therefore it might be involved in a pathway that acts early in the disease course of type 1 diabetes, leading to the anti-islet autoimmunity that we now know is established in most cases by the age of 3 years [9, 45].

A potential limitation of our study is that the majority (92%) of individuals with type 1 diabetes were diagnosed at less than 20 years old, and it is unlikely that we have identified all the variants associated with the AAD of type 1 diabetes. However, there is scope to perform a similar analysis in a population with more individuals diagnosed at over 18 years of age when data are generated in the future. Finally, some caution should be taken when interpreting the association at 1q24.3 (near *FASLG*), as the association did not reach a stringent genome-wide significance.

In conclusion, we have identified a novel AAD region at 6q22.33, as well as confirmed the well-established association of the MHC. The two risk haplotypes at 6q22.33 show evidence of association with type 1 diabetes in individuals diagnosed at less than 5 years of age and might thus guide therapeutic strategies in those with early-diagnosed type 1 diabetes.

## Electronic supplementary material

ESM (PDF 7898 kb)

## Abbreviations

AAD: Age at diagnosis
ASP: Affected sib-pair
FDR: False discovery rate
gMPPI: Group marginal posterior probability of inclusion
GRID: Genetic Resource Investigating Diabetes
GWAS: Genome-wide association study
IDDMGEN: Tyypin 1 Diabetekseen Sairastuneita Perheenjäsenineen
LD: Linkage disequilibrium
NI: Northern Ireland
T1DGC: Type 1 Diabetes Genetics Consortium
T1DGEN: Tyypin 1 Diabeteksen Genetiikka
