# Supplementary material for: The chromosome 6q22.33 region is associated with age at diagnosis of type 1 diabetes and disease risk in those diagnosed under 5 years of age
Source: Diabetologia. 2017 Oct 5;61(1):147–57. doi: 10.1007/s00125-017-4440-y (PMC5719131; doi:10.1007/s00125-017-4440-y)
Supplement: Supplementary file 1 — (PDF 7898 kb) [file 125_2017_4440_MOESM1_ESM.pdf]

Electronic Supplementary Material

Genotyping: Genotyping was performed in three separate batches, the first containing samples from UK GRID cases, the second on all T1DGC, and the third on all remaining cases (IDDMGEN, T1DGEN, Northern Irish GRID and WARREN cohorts).

Quality control: Quality control (QC) was performed separately by genotype batch. Individuals were excluded from the analysis if they had a low call rate or high heterozygocity rate, as these are indicators of poor genotyping. Individuals were also excluded if they had a very high pairwise identical by state proportion, as these would likely be duplicates. Finally, if an individual of a given sex had a very high or low homozygocity rate on their X-chromosome compared to that expected from that sex, they were excluded from the analysis. The per-individual cut-off values for excluding samples from the analysis were dictated by visual inspection of QC plots (Supplementary Figures 1, 2 and 3) rather than using set rules across all batches. Per-SNP QC was also performed, removing SNPs with >5% missing values and SNPs with a minor allele frequency (MAF) $<5\times10^{-5}$. Since Hardy-Weinberg Equilibrium (HWE) is important to check for in controls, we included SNPs that violated HWE but checked signal cloud plots at SNPs that were significantly associated with AAD using ImmunoBase (www.immunobase.org) to ensure the genotype groups were in distinct clusters.

ESM Table 1: Control patients included in meta-analysis examining type 1 diabetes risk overall and by age-strata

Cohort	Analysed against which cases	Type of controls	Number
T1DGC	T1DGC	Parents/siblings of T1DGC affected sib-pairs	6,282
IDDMGEN	IDDMGEN	Parents/siblings of IDDMGEN cases	2,390
WARREN	WARREN	Parents/siblings of WARREN affected sib-pairs	735
1958 British Birth Cohort	UK GRID	Independent controls	6,595
UK Blood Service	UK GRID	Independent controls	3,023
NI GRID	NI GRID	Independent controls	485
Total			19,510

ESM Table 2: Candidate causal variants AAD of type 1 diabetes in the 6q22.33 region, according to GUESSFM fine-mapping analysis for three, two and six SNPs expected in the region *a priori*. SNPs are colour-coded according to GUESSFM group.

Group	SNP	Position (GRCh37)	gMPPI when number expected SNPs in model=3	gMPPI when number expected SNPs in model=2	gMPPI when number expected SNPs in model=6
1	rs802750	128265919	0.497	0.488	0.55
	rs802747	128268565			
	rs802744	128272324			
	rs802743	128272876			
	rs802740	128276700			
	rs802739	128277210			
	rs802738	128277933			
	rs802737	128278053			
	rs376827043	128278122			
	rs1418600	128278230			
	rs1418601	128278231			
	rs802735	128278336			
	rs802733	128279185			
	rs802732	128279422			
	rs35576497	128279497			
	rs802728	128281556			
	rs802727	128281661			
	rs802726	128281861			
	rs1089652	128282783			
	rs802724	128283193			
	rs802722	128284219			
	rs802721	128284771			
2	rs802746	128269180	0.416	0.416	0.463
	rs802734	128278798			
	rs802731	128279429			
	rs802730	128280104			
	rs802725	128282029			
	rs1089653	128282758			
	rs802719	128289019			
	rs3190930	128291199			
	rs41285280	128291649			
	rs4559105	128292392			
	rs55743914	128293562			
	rs35469349	128294709			
3	rs6939352	128266250	0.962	0.962	0.95
	rs9491889	128270067			
	rs9491890	128270123			
	rs9491891	128277151			
	rs147626184	128277275			
	rs118097399	128278233			
	rs9491892	128280358			
	rs9482848	128280375			
	rs9491893	128280931			
	rs113297984	128286301			
	rs72973797	128286386			
	rs72973800	128287158			
	rs761332	128287848			
	rs9482849	128288536			
	rs12111314	128289214			
	rs11753289	128291681			
	rs9482850	128293506			
	rs9482851	128293634			
	rs72975913	128293932			
	rs72975916	128294055			

	rs7738609	128295502
	rs138300818	128297022
	rs3901020	128297604
	rs4510698	128297611

ESM Table 3: Results of type 1 diabetes risk in those diagnosed at a young age, using different cut-offs to define the youngest AAD strata

**SNP**	**Cut-off**	**N cases**	**OR (95% CI)**	***p*-value**
rs72975913	<5	3,807	0.78 (0.72 - 0.85)	2.32e-09
	<4	2,846	0.75 (0.68 - 0.82)	1.01e-09
	<6	4,758	0.80 (0.74 - 0.86)	3.8e-09
rs802719	<5	3,806	1.14 (1.07 - 1.20)	2.23e-05
	<4	2,846	1.13 (1.06 - 1.21)	1.98e-04
	<6	4,757	1.12 (1.06 - 1.18)	5.06e-05

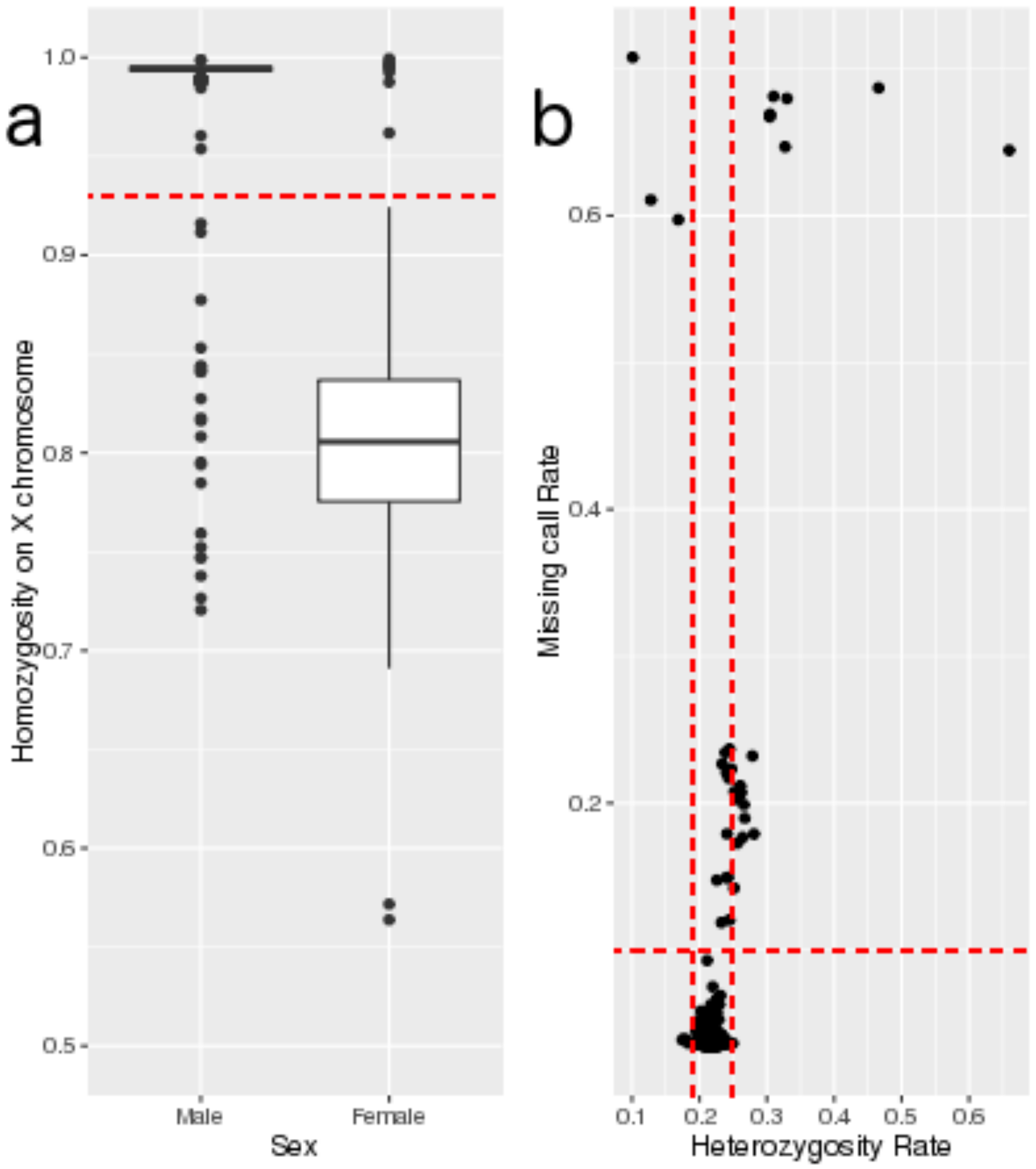

ESM Figure 1: Quality control plots for the UK GRID dataset. Panel a) shows the homozygosity rate on the X chromosome by sex, where one would expect males to have most alleles as homozygous. Low homozygosity rate in males or homozygosity rate close to 1 in females indicates poor genotyping or sample swaps and therefore samples are excluded from the analysis. Panel b) shows individuals with particularly high missingness or heterozygosity rate across the non-sex chromosomes, for which individuals are excluded from the analysis. Samples outside the red dashed lines were excluded.

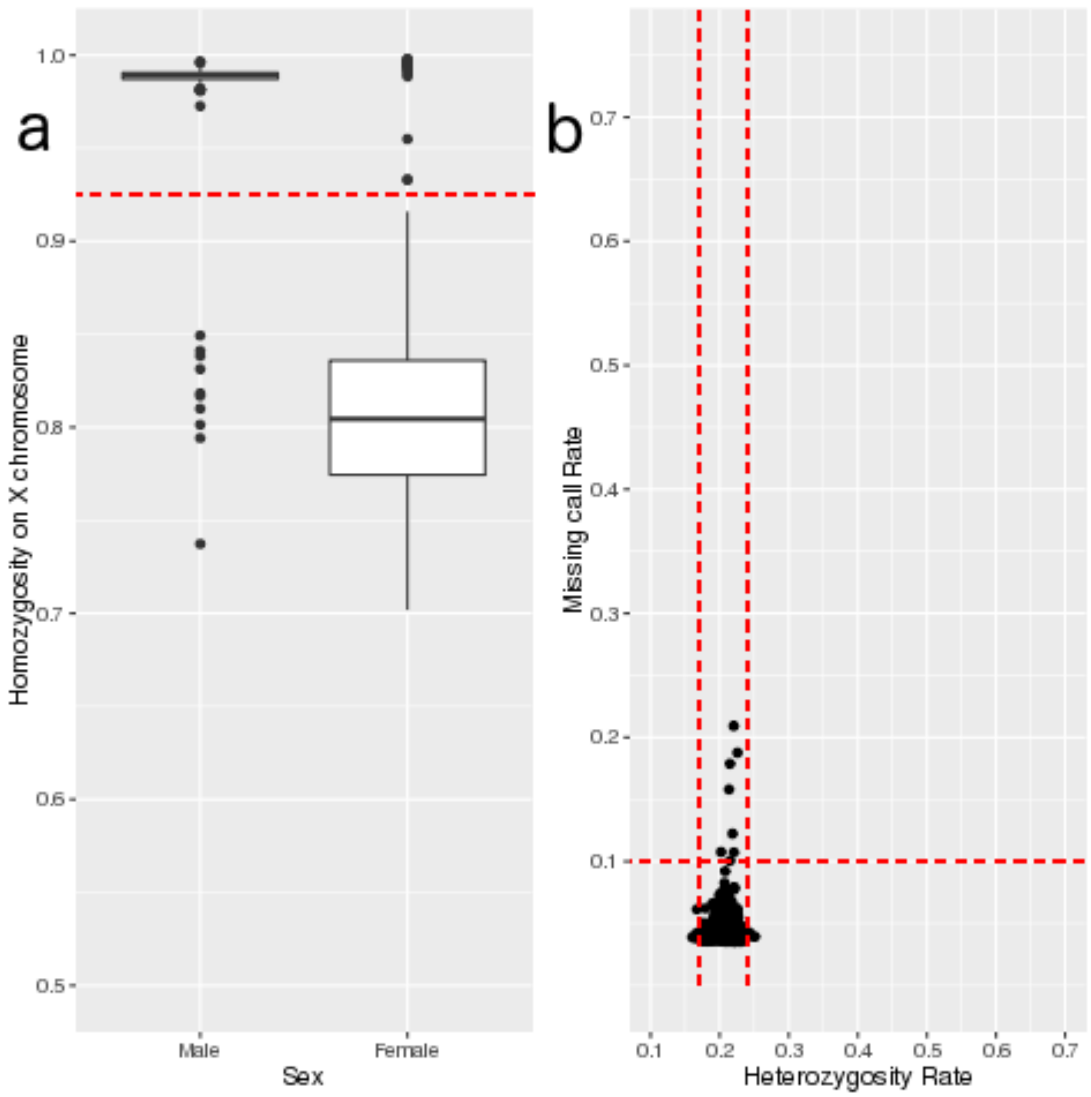

ESM Figure 2: Quality control plots for the T1DGC affected sib-pairs dataset. Panel a) shows the homozygosity rate on the X chromosome by sex, where one would expect males to have most alleles as homozygous. Low homozygosity rate in males or homozygosity rate close to 1 in females indicates poor genotyping or sample swaps and therefore samples are excluded from the analysis. Panel b) shows individuals with particularly high missingness or heterozygosity rate across the non-sex chromosomes, for which individuals are excluded from the analysis. Samples outside the red dashed lines were excluded.

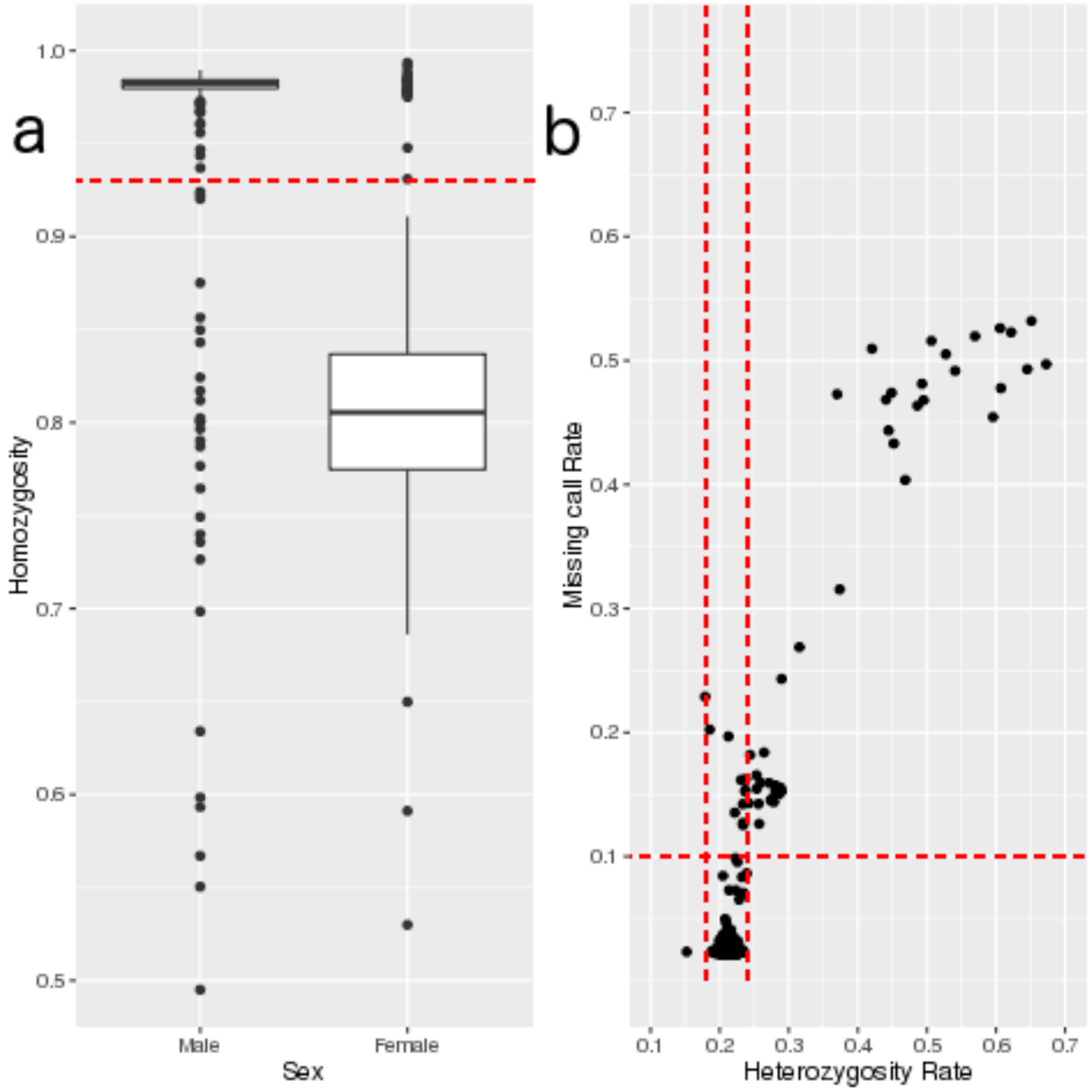

ESM Figure 3: Quality control plots for the dataset containing the Northern Irish GRID cases, Finnish IDDMGEN and T1DGEN cases and UK WARREN cohort affected sib-pairs. Panel a) shows the homozygosity rate on the X chromosome by sex, where one would expect males to have most alleles as homozygous. Low homozygosity rate in males or homozygosity rate close to 1 in females indicates poor genotyping or sample swaps and therefore samples are excluded from the analysis. Panel b) shows individuals with particularly high missingness or heterozygosity rate across the non-sex chromosomes, for which individuals are excluded from the analysis. Samples outside the red dashed lines were excluded.

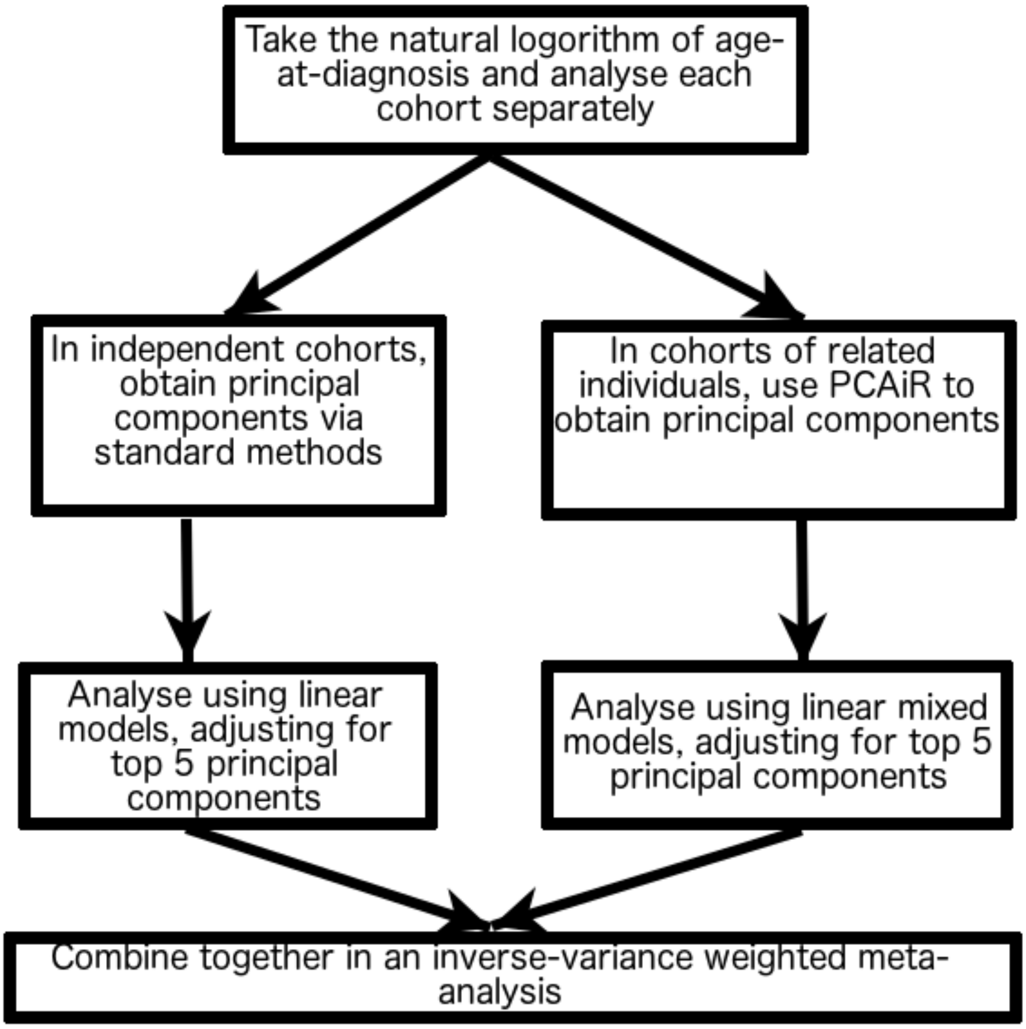

ESM Figure 4: Schematic diagram showing analysis pipeline for meta-analysis in the variant discovery analysis for age-at-diagnosis of type 1 diabetes.

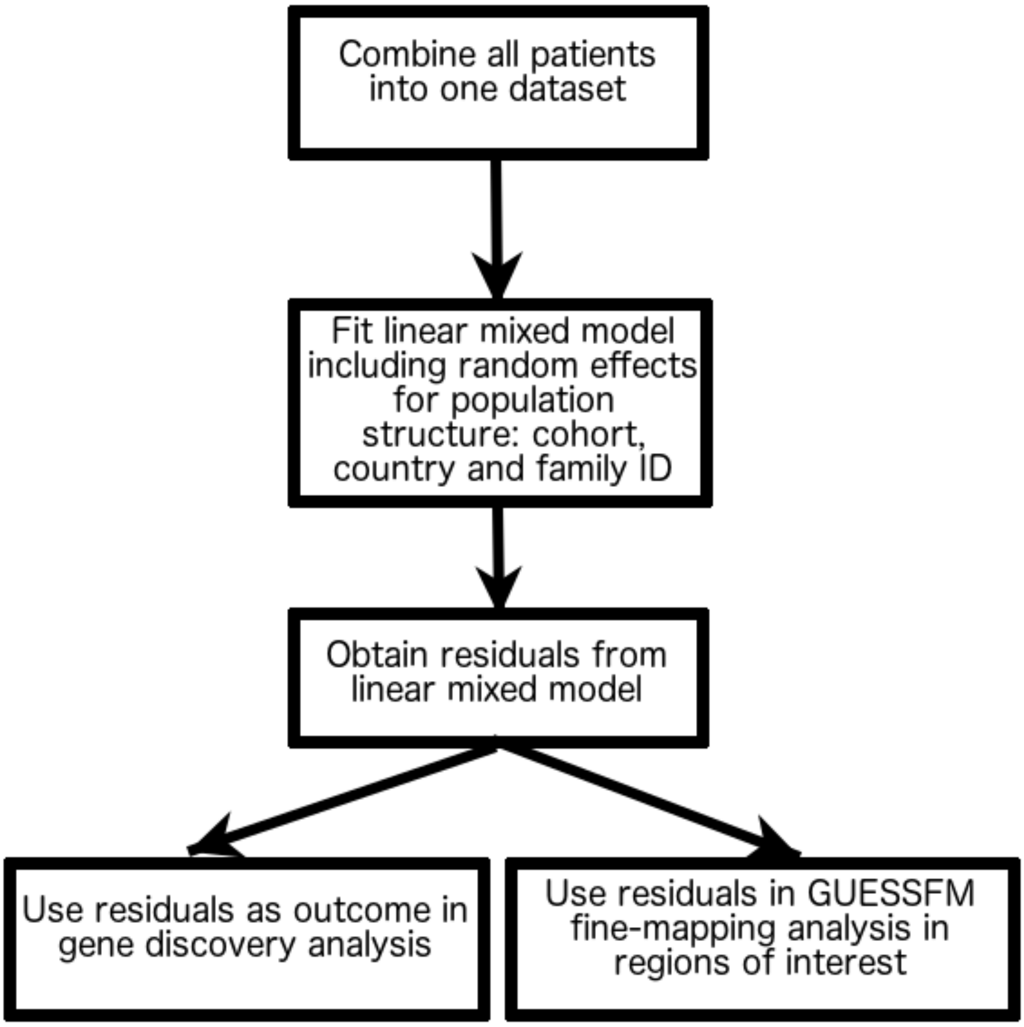

ESM Figure 5: Schematic diagram showing analysis pipeline for the residual-based model variant discovery analysis and fine-mapping of associated regions.

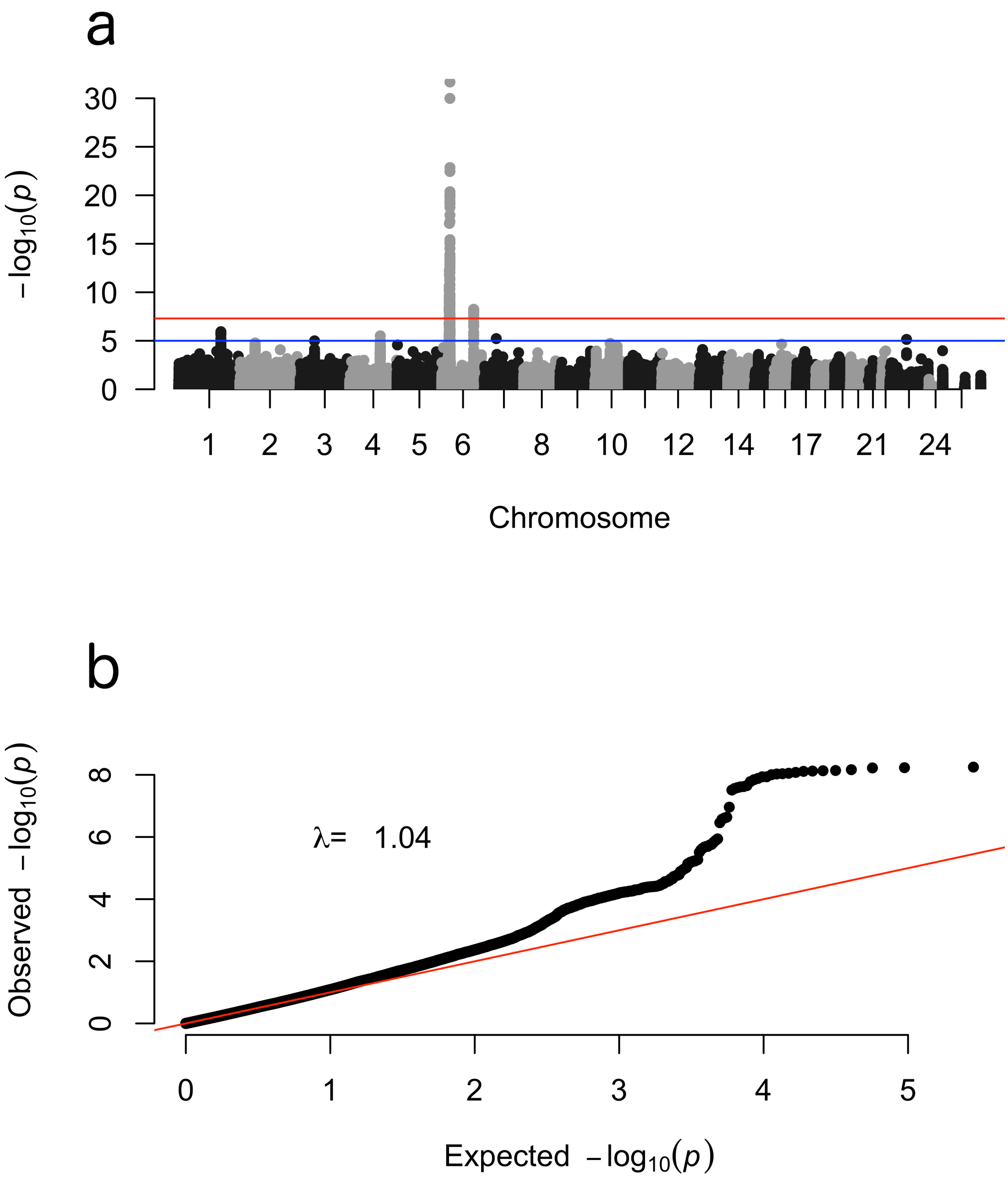

ESM Figure 6: Panel a) Manhattan plot and panel b) QQ plot (excluding the MHC region) for the residual-based model, examining genetic determinants of AAD of type 1 diabetes, with cut-off significance lines at $–\log_{10}p = 5.0$ (blue) and $–\log_{10}p = 8.0$ (red).

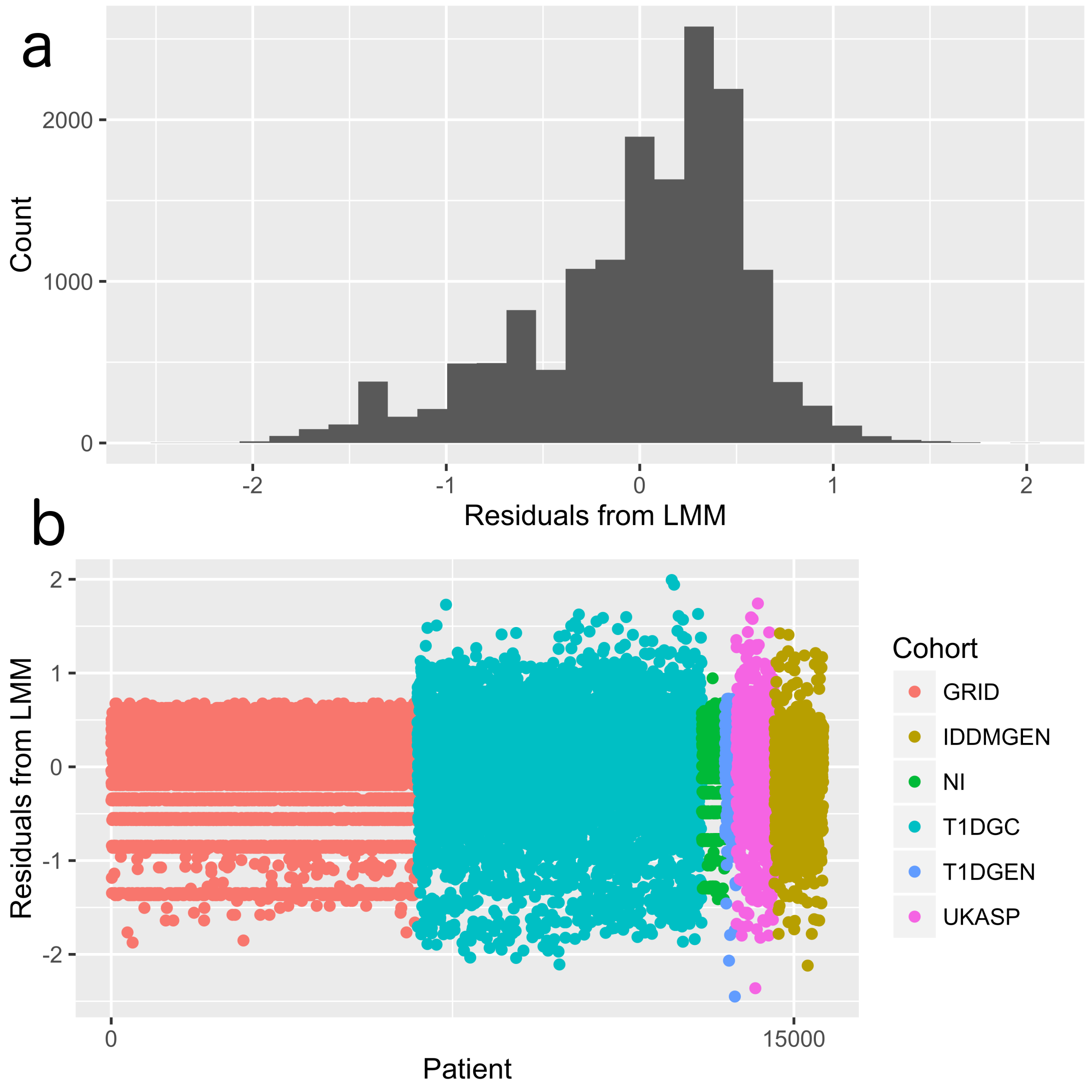

ESM Figure 7: Residual plots from the linear mixed model, with the natural logarithm of AAD as the outcome, adjusting for the fixed effect of sex and the random effects of country, cohort and family identifier. Panel a) shows a histogram of the residuals and panel b) shows the residuals plotted by individual, colour-coded by what cohort they are from.

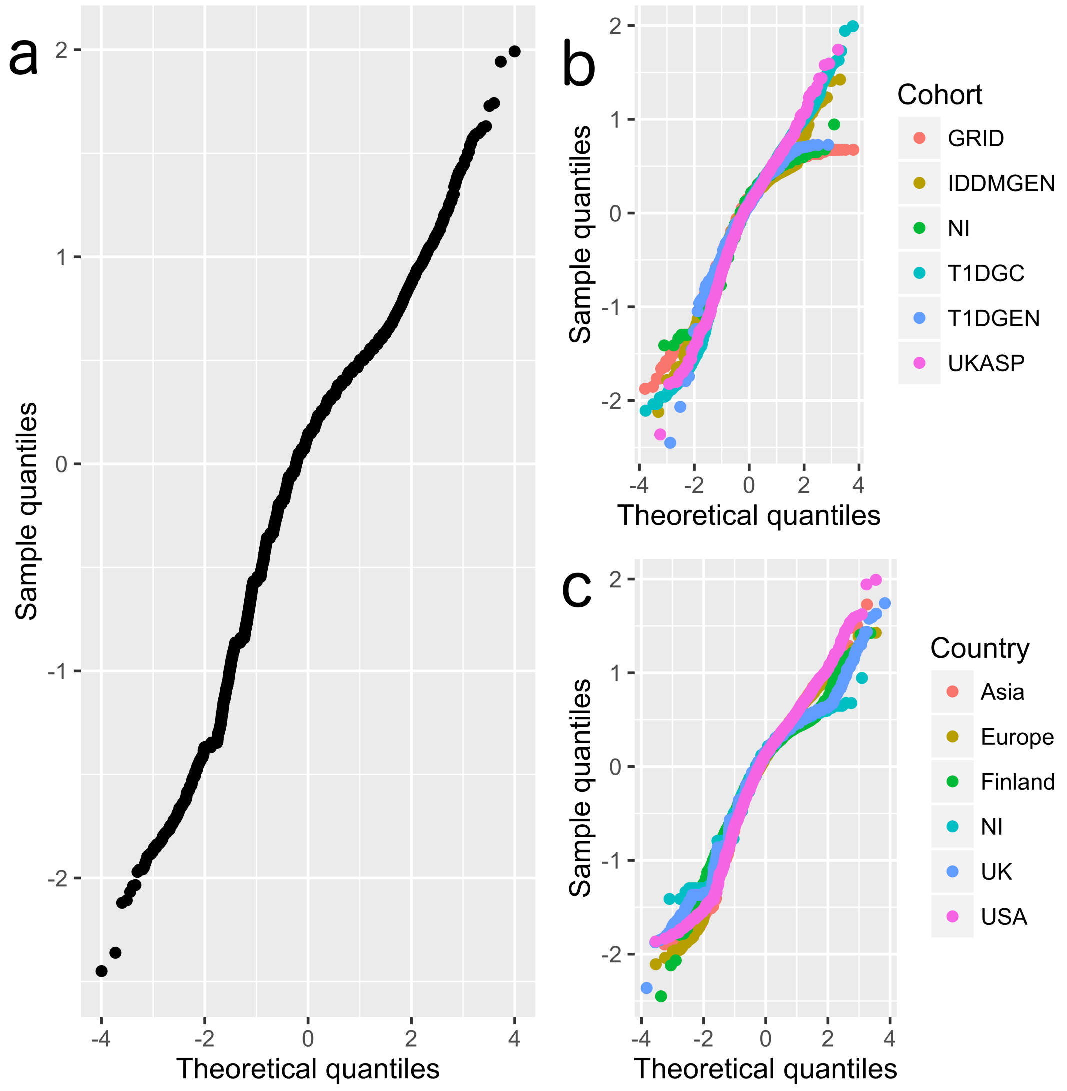

ESM Figure 8: Residual QQ plots from the linear mixed model, with the natural logarithm of AAD as the outcome, adjusting for the fixed effect of sex and the random effects of country, cohort and family identifier. Plot a) shows the QQ plot for all individuals in the dataset, whilst plots b) and c) are stratified by cohort and country, respectively.

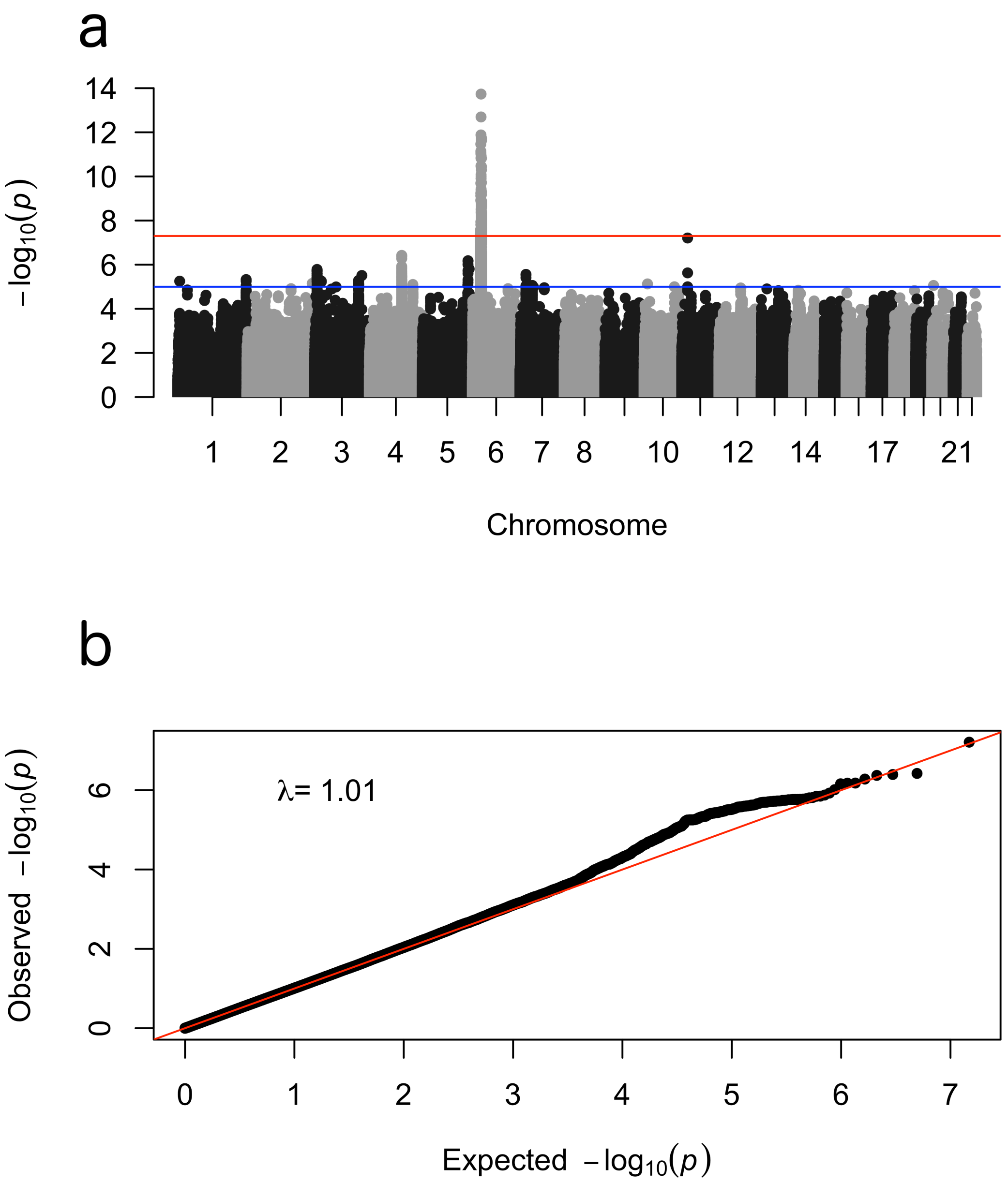

ESM Figure 9: Panel a) Manhattan plot and panel b) QQ plot (excluding the MHC region) for AAD of type 1 diabetes in UK GRID cases, imputing SNPs genome wide and meta-analysed combining two subsets of the GRID cohort, one that was genotyped using Affymetrix technology (N=1,768) and the other that was genotyped using the Illumina technology (N=3,833). Significance cut-off lines are at $-\log_{10}p$ = 5.0 (blue) and $-\log_{10}p$ = 8.0 (red).

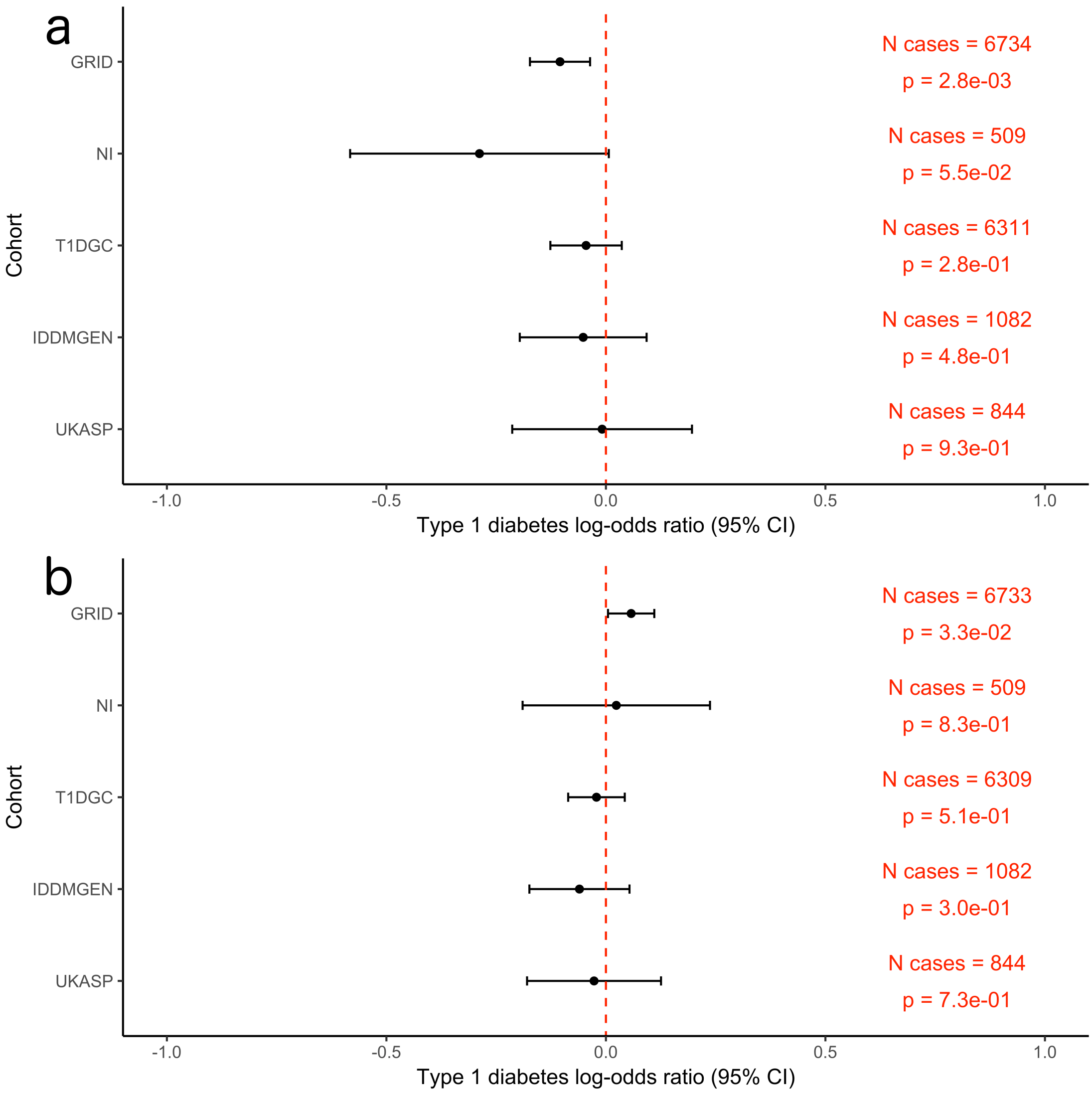

ESM Figure 10: Forest plot showing the effect of rs72975913 (panel a)) and rs802719 (panel b)) on type 1 diabetes risk overall in each cohort.

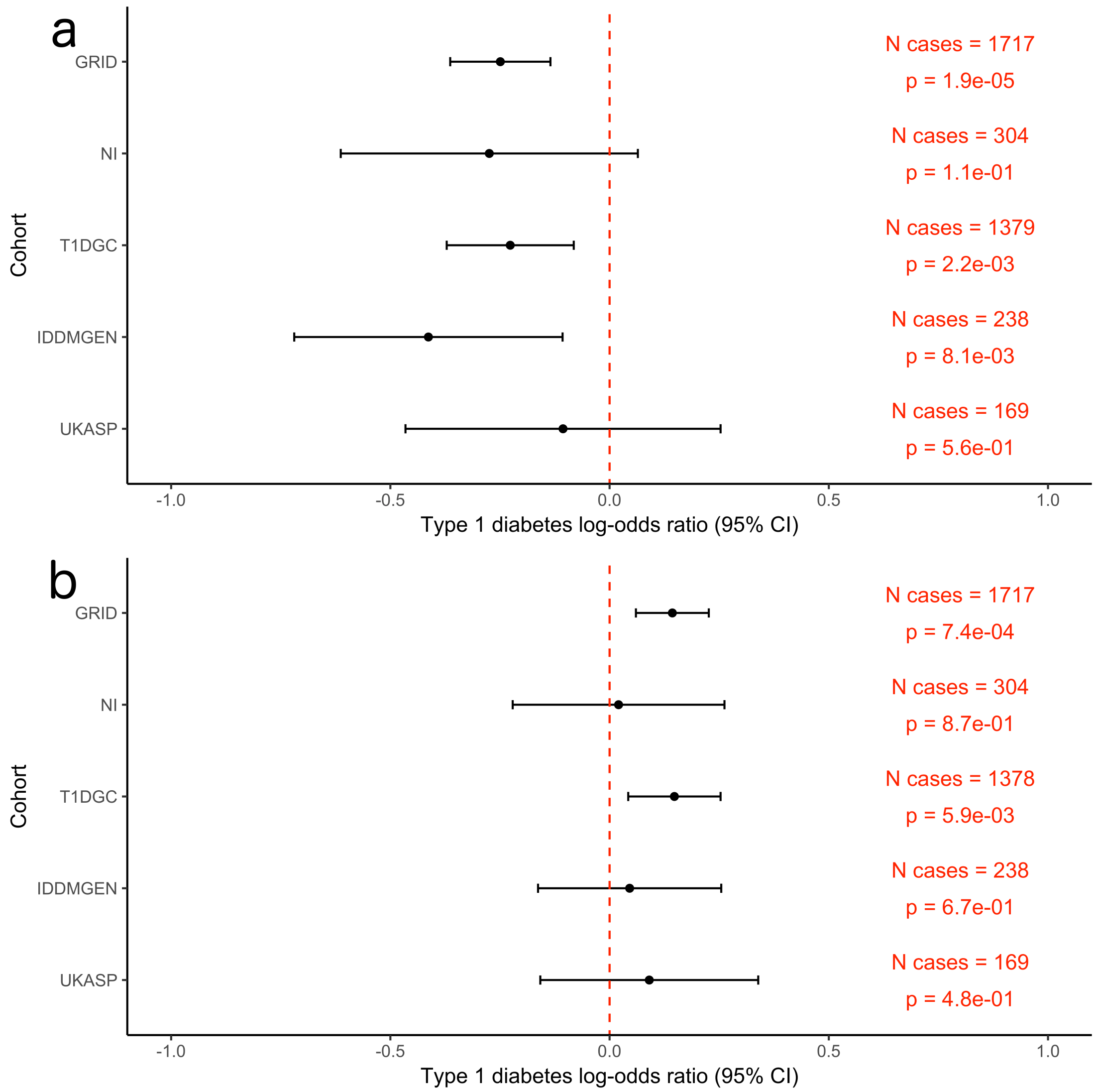

ESM Figure 11: Forest plot showing the effect of rs72975913 (panel a)) and rs802719 (panel b)) on type 1 diabetes risk in individuals diagnosed at <5 years old in each cohort.

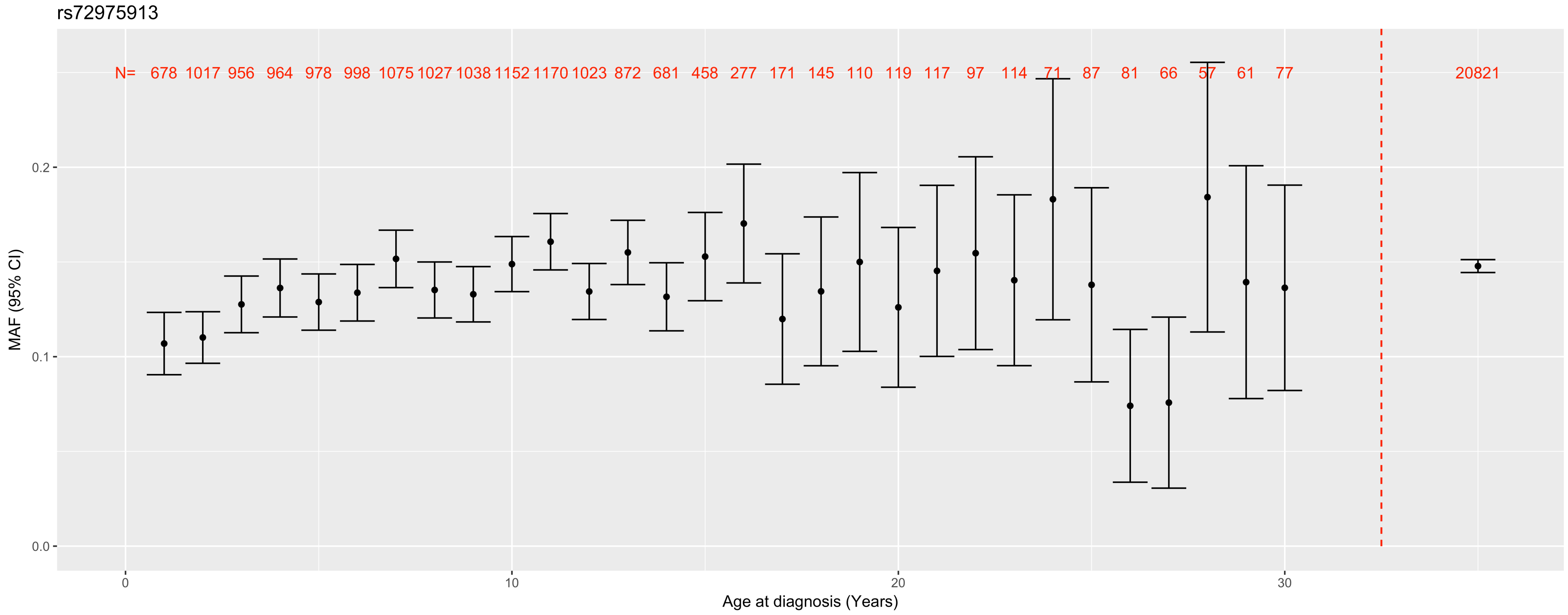

ESM Figure 12: Minor allele frequencies by age at diagnosis at rs72975913, compared with controls to the right of the dashed red line.

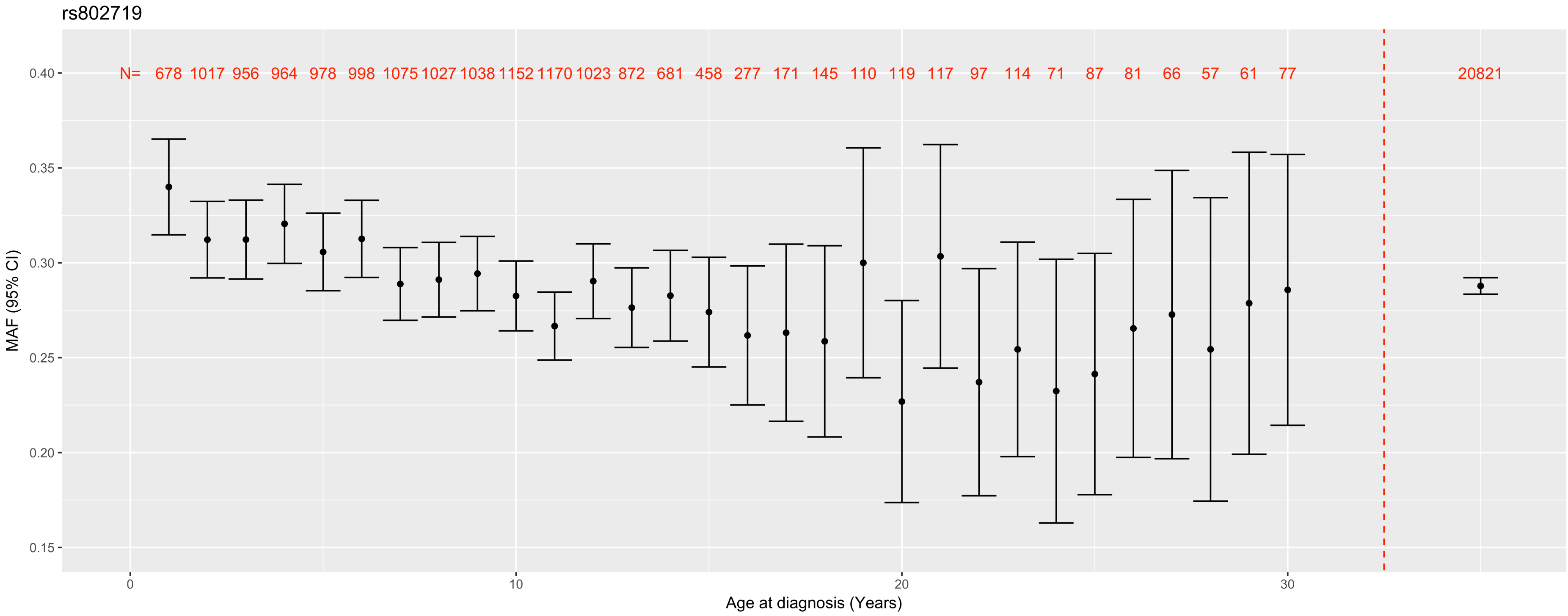

ESM Figure 13: Minor allele frequencies by age at diagnosis at rs802719, compared with controls to the right of the dashed red line.
